# What Lies Ahead for Young Hearts in the 21^st^ Century – Is It Double Trouble of Acute Rheumatic Fever and Kawasaki Disease in Developing Countries?

**DOI:** 10.3389/fcvm.2021.694393

**Published:** 2021-06-24

**Authors:** Aaqib Zaffar Banday, Sanjib Mondal, Prabal Barman, Archan Sil, Rajni Kumrah, Pandiarajan Vignesh, Surjit Singh

**Affiliations:** Allergy Immunology Unit, Department of Pediatrics, Advanced Pediatrics Centre, Post Graduate Institute of Medical Education and Research, Chandigarh, India

**Keywords:** epidemiology, fever, heart, incidence, Kawasaki disease, rheumatic, trend

## Abstract

Rheumatic heart disease (RHD), the principal long-term sequel of acute rheumatic fever (ARF), has been a major contributor to cardiac-related mortality in general population, especially in developing countries. With improvement in health and sanitation facilities across the globe, there has been almost a 50% reduction in mortality rate due to RHD over the last 25 years. However, recent estimates suggest that RHD still results in more than 300,000 deaths annually. In India alone, more than 100,000 deaths occur due to RHD every year (Watkins DA et al., N Engl J Med, 2017). Children and adolescents (aged below 15 years) constitute at least one-fourth of the total population in India. Besides, ARF is, for the most part, a pediatric disorder. The pediatric population, therefore, requires special consideration in developing countries to reduce the burden of RHD. In the developed world, Kawasaki disease (KD) has emerged as the most important cause of acquired heart disease in children. Mirroring global trends over the past two decades, India also has witnessed a surge in the number of cases of KD. Similarly, many regions across the globe classified as “high-risk” for ARF have witnessed an increasing trend in the incidence of KD. This translates to a double challenge faced by pediatric health care providers in improving cardiac outcomes of children affected with ARF or KD. We highlight this predicament by reviewing the incidence trends of ARF and KD over the last 50 years in ARF “high-risk” regions.

## Introduction

Globally, acute rheumatic fever (ARF) remained the most important cause of acquired heart disease in children until the later part of the 20th century. Improvements in general standard of living, hygiene, sanitation, health care facilities, better understanding of the disease, appropriate use of antimicrobials, and directed public health policies resulted in a significant decrease in incidence of ARF and prevalence of rheumatic heart disease (RHD) in developed countries (1, 2). On the other hand, Kawasaki disease (KD) is increasingly being recognized in many developed and developing countries. KD is now the commonest cause of acquired heart disease in children in developed countries and the incidence of KD in developing countries also seems to be rising (3).

However, in many under-developed and developing regions, a significant burden of ARF/RHD still exists due to poor standard of living, suboptimal hygiene and sanitation facilities (4). Also, KD has clinical features overlapping with many infectious diseases and no specific diagnostic tests for KD are available. It is possible that children with KD in developing countries may end up being empirically treated with antimicrobials given the tremendous burden of infectious diseases in these countries (5). Additionally, timely intravenous immunoglobulin therapy remains a challenge due to concerns of limited availability and high costs involved in its procurement. Consequently, a higher proportion of children with KD in developing countries may suffer cardiac complications (as compared to the developed world) (6–10).

In this review, we note the trends in the incidence of ARF and KD [before the severe acute respiratory syndrome coronavirus 2 (SARS-CoV-2) pandemic] in regions that have been traditionally described to have “high-risk”/“high-burden” for ARF/RHD. We highlight that the incidence of KD is increasing in these areas while a significant burden of RHD remains. So there is a dual challenge (of KD and ARF) in a majority of regions in the developing world that needs to be tackled to reduce the burden of cardiovascular morbidity in children.

## History (Including Diagnostic Criteria)

### ARF

The first descriptions indicative of acute rheumatic fever (ARF) and its cardiac sequel date back to the first century A.D (11, 12). Sydenham in late 1600s provided a detailed description of ARF and, for the first time in history, he differentiated this illness from gout (13). The first probable description of ARF in modern English language was published around 1700 (14). Around the turn of the 20th century, the complete spectrum of ARF in the pediatric population was described (15, 16). The year 1944 was a landmark in history of rheumatism when Dr. T. Duckett Jones, for the first time, proposed diagnostic criteria for ARF (17, 18). The Jones criteria have been revised on numerous instances with the latest version having been published in 2015 (19, 20). The latest version incorporates monoarthralgia as a minor criterion, and monoarthritis and polyarthralgia as major manifestations in high-risk settings. Additionally, lower cutoffs for fever and erythrocyte sedimentation rate have been promulgated for such populations (Supplementary Table 1). These substantial revisions will enhance the recognition of ARF in high-risk populations across the globe (20).

### KD

The first descriptions of Kawasaki disease (KD) were published by Dr. Tomisaku Kawasaki in 1967 and 1974 in Japanese and English language, respectively (21–23). In 1975, Kato et al. published the first English language description of coronary artery aneurysms in KD detected by coronary angiography (24, 25). Two sets of diagnostic criteria are currently employed for KD – the American Heart Association (AHA) and the Japanese criteria. These diagnostic criteria are essentially based on the seminal observations made by Dr. Kawasaki in the 1960s. The latest version of the AHA diagnostic criteria has been published in 2017 (3) and the latest version of Japanese criteria has been published in the English Language in 2020 (26). Essentially, both these sets of criteria incorporate fever, rash, bilateral non-exudative conjunctival injection, oral mucosal changes, cervical lymphadenopathy, and edema or periungual skin peeling of hands or feet as major manifestations of the disease (Supplementary Tables 2, 3). However, clinical judgment is imperative as KD may present only with fever and coronary artery abnormalities, especially in young infants (3). The recent Japanese criteria have been updated to augment the diagnosis of KD – for example, reactivation of Bacillus Calmette–Guérin vaccination site, most commonly seen infants and young children, has been added as a major manifestation (26). Similar modifications to the AHA criteria may increase the sensitivity of KD diagnosis. Analogously, revisions have already been made by the AHA to the modified Jones criteria (in 2015).

## Trend of Incidence

### India

#### ARF

**Figure 1:**
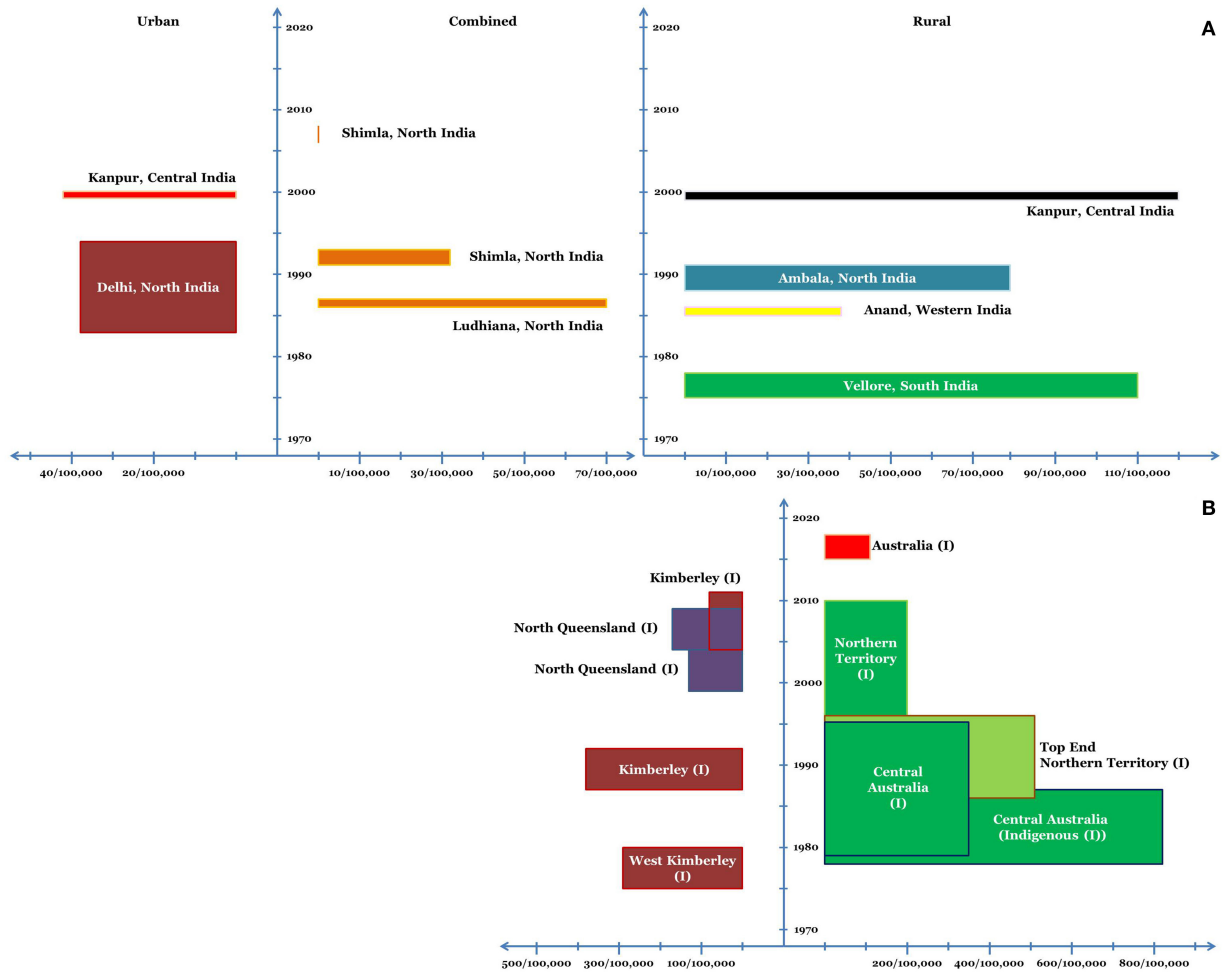
Incidence of ARF in various regions of India **(A)** and Australia **(B)** in school-age children. (I) refers to the indigenous population. The x-axis depicts the incidence and the y-axis depicts the timeline. The dimension of the bars along the x-axis (length of the bars) depicts the average annual incidence of ARF. The dimension of the bars along the y-axis (width of the bars) corresponds to the duration for which the said average annual incidence was noted.

In a school-based study from rural South India in 1970s, the average incidence of rheumatic fever and newly diagnosed rheumatic heart disease was estimated to be 110/100,000 schoolchildren per year (27). In a population-based study from rural North India in late 1980s, the average incidence of first episode of ARF in children 5–15 years of age was estimated to be 54/100,000 per year (overall ~79/100,000) (28). Surveys conducted in 1980s and early 1990s estimated the annual incidence of ARF in pediatric age group to be 38–70/100,000 (29). In early 1990s, a study from North India estimated the annual incidence of first episode of ARF to be 19/100,000 school children (overall 32/100,000) (30). In a North Indian survey of ~16,000 children between 5–15 years of age, no cases of ARF were reported during the observation period of 2007 and 2008 (29). Multicentric studies have noted a declining trend in the prevalence and burden of RHD in India; however, studies on the incidence of ARF are sparse (Figure 1) (31).

#### KD

**Figure 2:**
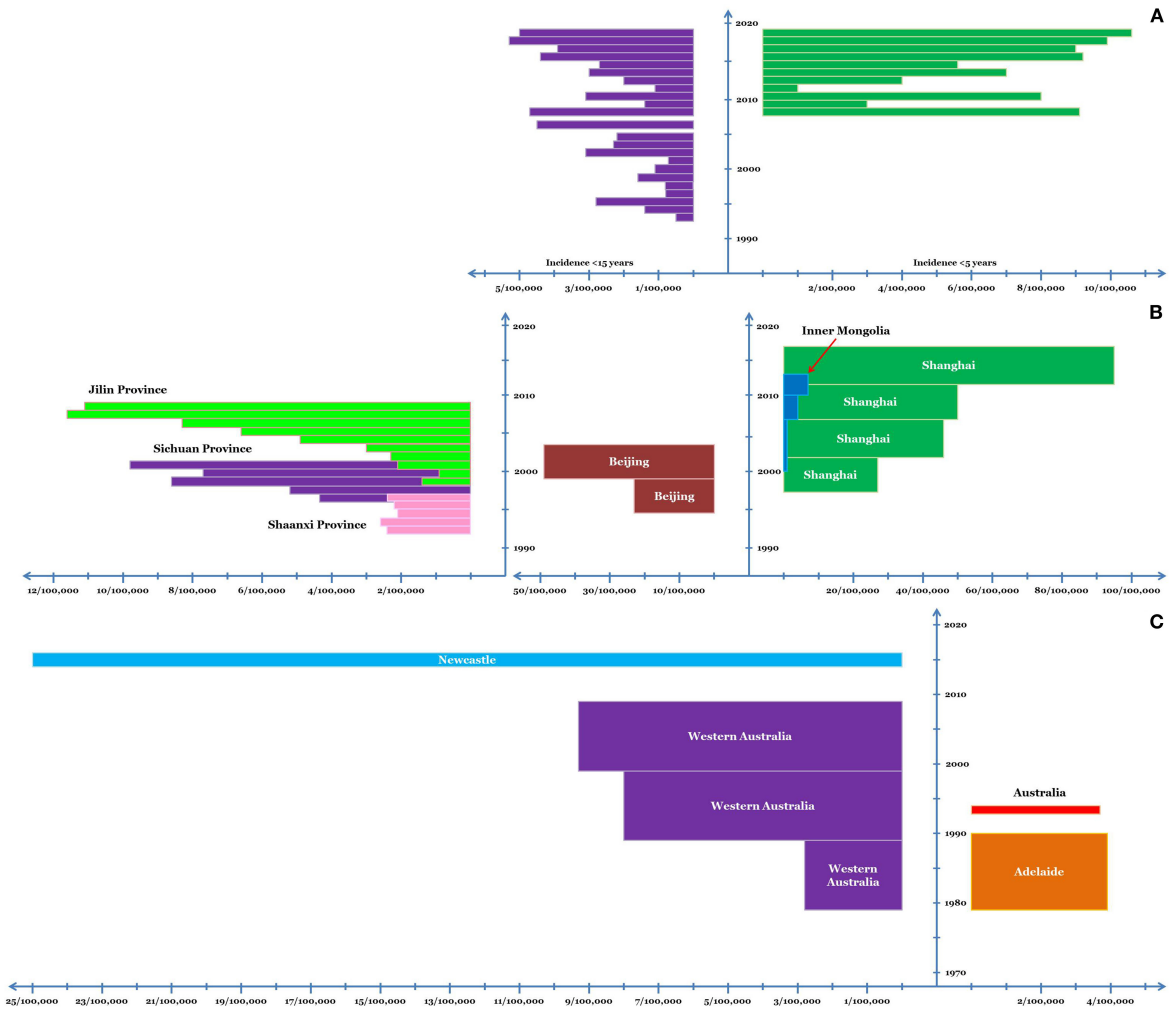
Incidence of KD in Chandigarh, India, **(A)** and various regions of China **(B)** and Australia **(C)**. In case of Chandigarh, India, the incidence in children <5 and <15 years of age is shown; while for China and Australia, incidence in young children is depicted. The x-axis depicts the incidence and the y-axis depicts the timeline. The dimension of the bars along the x-axis (length of the bars) depicts the average annual incidence of KD. The dimension of the bars along the y-axis (width of the bars) corresponds to the duration for which the said average annual incidence was noted.

Prior to 1990, there were only three reports of KD from India. Subsequently, hospital-based incidence studies were undertaken predominantly from Chandigarh, North India (32). In children below 15 years, the incidence of KD was estimated to be 0.51/100,000 in 1994. The incidence gradually increased in the subsequent years and was estimated to be 4.54/100,000 in 2007 (33). In children <5, the average annual incidence of KD during 2009–14 was 5.35/100,000 (34). The incidence of KD in children <5 years at Chandigarh has been estimated to be 5.64/100,000 and 10.6/100,000 during 2015 and 2019, respectively (unpublished observations) (Figure 2). Other centers in India have also witnessed a similar increase in the number of cases with KD in the last decade, although nationwide estimates for incidence are lacking (35–37).

### China

#### ARF

English language reports on incidence of ARF in China are scarce. In early 1990s, the annual incidence of ARF in the pediatric population in South Western China was estimated to be 12.87/100,000 (38). In a large survey across five provinces in mainland China conducted between 1992–1995, the average annual incidence of ARF in children 5–18 years of age was reported to be 20.05/100,000 (39, 40). Although population-based data reflecting the trend of ARF incidence in China are sparse, hospital-based data have noted a decreasing trend similar to other regions of the world (39, 41).

#### KD

##### Shanghai and Beijing

A number of studies have been conducted in China to assess the epidemiology of KD. By the turn of the 21st century, KD had already overtaken ARF in the annual incidence, at least in Shanghai and Beijing. In Shanghai, the average annual incidence of KD in children below five has gradually increased over the last two decades which was 27.32/100,000 during 1998–2002, 46.32/100,000 during 2003–07, 50.5/100,000 during 2008–12, and 94.7/100,000 during 2013–17 (42–44). In Beijing, the average annual incidence of KD has been similar to Shanghai; for example, during 1995–99 it was 22.9/100,000 (45), while during 2000–04 it was 49.4/100,000 (46). However, reports suggest significant variability in the incidence of KD among different regions in China.

##### Other Regions

The average annual incidence of KD in Jilin Province during 1999–2008 was 5.04/100,000 children under five (47). Similarly, in Inner Mongolia, the mean annual incidence of KD during 2001–13 was 3.55/100,000 children under 5 (48). Nonetheless, these surveys did note an increasing trend in the disease incidence during these periods. In Shaanxi province, the annual incidence of KD remained fairly constant at ~2.34/100,000 children <5 years of age during mid-1990s (49). However, an increasing trend in the incidence of KD was noted in Sichuan Province where the incidence of KD in children <5 years was 4.26/100,000 and 9.81/100,000 in 1997 and 2001 respectively (50) (Figure 2).

### Australia

#### ARF

##### Western Australia

In the late 1970s, hospital-based studies from West Kimberley (Western Australia) estimated the annual incidence of ARF to be 230–350/100,000 in Indigenous school children (51). During 1988–92, the annual incidence of ARF in Indigenous school-children (5–14 years) in the Kimberley region was 375/100,000 (52). The annual incidence in Indigenous population in the age group of 15–29 years was also high at 258/100,000 (52). Hospital-based incidence data, however, suggested a decrease in incidence of ARF in the region. During 1988–92, the annual incidence as per 100,000 hospitalized school-children was 278 (52). The annual age-standardized hospitalization rates for ARF during 2005–11 ranged between 50–100/100,000 Indigenous population in Kimberley (except for 2008, when it was <50/100,000) (53). In the same study, a significant yearly decrease of ~9% in the age-standardized hospitalizations of ARF/RHD was noted during 2003–11 (continued decrease noted for both ARF and RHD) (53, 54).

##### Queensland

The annual incidence of ARF in Indigenous children (5–14 years) in north Queensland during 1999–2004 was 133/100,000. The highest incidence was noted in Northern Peninsula Area and Torres Strait Health Service District (349/100,000 children), whereas, no ARF case was detected in Innisfail District (55). A significant increase in the incidence of ARF was noted over the next 5 years (2004–09) with the annual incidence in children 5–14 years of age being 155/100,000. This finding also reflected that the transition from intensified to usual surveillance in the year 2004 had no adverse bearing on the notification of ARF (56).

##### Northern Territory

During 1978–87, the incidence of ARF in Central Australian Indigenous children (5–14 years) was estimated to be 815/100,000 (57). From 1987–96, the incidence in Indigenous children in Top End Northern Territory was estimated to be 224/100,000. However, the incidence in Indigenous communities where complete data was available was higher at 508/100,000 and in Non-Indigenous children (5–14 years) the incidence was 1.3/100,000 (58). In a combined study assessing the epidemiology of ARF in Northern Territory during 1979–96, the incidence of ARF in 5–14 year old indigenous children in Top End and Central Australia was estimated to be 245/100,000 and 351/100,000 respectively (59). During 1997–2010, the annual incidence of the first episode of ARF in the Indigenous children (5–14 years) of the Northern Territory was 194/100,000 (60). Notably, in this large and well-designed study, a decreasing trend in the annual incidence of ARF was not noted on multivariate analysis (60).

The annual incidence of the first episode of ARF across 5 Australian Jurisdictions during 2015–2017 was 107.6/100,000 for Indigenous children (5–14 years) and 1/100,000 for non-Indigenous children (61). More than 80% of the ARF episodes were reported from North Australia (61) (Figure 1).

#### KD

During 1979–90, the average annual incidence of KD in Adelaide was 3.9/100,000 children aged 0–5 years, with highest incidence being noted in the year 1986 (7.7/100,000) (62). In 1994, the incidence of KD in Australian children <5 years was noted to be 3.7/100,000 (63). A large study analyzing the 30-year epidemiology of KD in Western Australia from 1979–2009 noted a gradual increase in the incidence of KD in children <5 years of age. The average annual incidence rate for the said population during the consecutive decades of the study was 2.82, 7.96, and 9.34 per 100,000 respectively (64). A hospital-based study from Newcastle estimated the average annual incidence of KD (in children <5 years) to be ~25/100,000 during 2015–16 (65) (Figure 2). In a nationwide study, an increase in the hospitalizations due to KD (0–19 years) was noted during the 25-year period from 5.2/100,000 in early 1990s to 12.4/100,000 in 2017–18 (66). Notably, very few Indigenous children have been diagnosed to have KD in these large studies (64, 66).

### Africa

#### ARF

Most of the studies have focused on the prevalence of RHD from Africa; however, epidemiological studies assessing the incidence of ARF are quite scarce. It has been estimated that half of the global RHD population under the age of 15 years lives in Africa (67). In the year 1990, the annual incidence of ARF in school-going children was estimated to be 30/100,000 (68). In Algerian children and adolescents (aged 4–19 years), the annual incidence of ARF was estimated to be 11.1/100,000 in 1997 and 6.2/100,000 in 2000 (69). A systematic review on the global burden of group A streptococcal disease by Carapetis et al. has noted a lesser incidence of ARF in sub-Saharan and North Africa in comparison to other “high-risk” regions (70). Poor case ascertainment and documentation seem to be the likely reasons for the lesser incidence of ARF in Africa (70).

#### KD

Similar to ARF, the actual incidence of KD in many African countries is not known. Variable incidence rates amongst children below 5 years of age have been documented from different African countries, for example, 3.15/100,000 from Algeria, 0.95/100,000 from Tunisia, and 4.52/100,000 from Morocco (71). Although incidence studies of KD are largely lacking, the increasing number of publications from Africa are encouraging and portend enhanced detection of KD in near future (7, 8).

### Latin America

#### ARF

The highest population-based annual incidence of ARF (~360/100,000 children aged 10–20 years) in Latin America has been documented in Belo Horizonte, Brazil during the year 1992 (69, 72). However, the study was carried out in a limited population only and the duration of the study was from March to December 1992 (72). Other studies from the region have noted at least a 5-fold lower incidence of ARF. In Cuban province of Pinar del Rio, a marked reduction in incidence of ARF (in the age group 5-25 years) was noted during 1986 to 1996 wherein the incidence rate decreased from 18.6/100,000 to 2.5/100,000, respectively (73). In the French Caribbean islands of Martinique and Guadeloupe, the incidence of ARF (in the age group of <20 years) in 1982–83 was 19.6/100,000 and 17.4/100,000, respectively. By 1992, the incidence was reduced by about 4- to 5-fold in both the regions as a result of a decade-long educational program (74). A study from Chile has noted a gradual decline in the incidence of ARF from 3/100,000 inhabitants in 1979 to 0 in 1998 (75). During 1994–99, a hospital-based study from Mexico noted incidence of first episode of ARF to be 660/100,000 cases admitted to the hospital (700/100,000 children and adolescents aged 5–20 years) (76). The incidence of ARF during this period was noted to be lower than the early 1970s when it was 1060/100,000 subjects (76, 77).

#### KD

The exact incidence of KD in many Latin American countries remains unknown. For precise estimation of disease burden, a research network was officially formed in 2013 comprising of 20 countries (78). Using the nationwide hospital-based data, the incidence of KD in Chilean children <5 years of age was estimated to be 5.7/100,000 in 2001–2004, 8.4/100,000 in 2005–2007, and 10.4/100,000 in 2009–2011 (79, 80). The highest incidence of KD (19.8/100,000) was noted in Eastern Metropolitan Health District region that, notably, had the highest socioeconomic status in the country (80).

The trends in the incidence of KD and ARF in regions classified as “high-risk” for ARF are summarized in Supplementary Tables 4, 5.

## Discussion

KD and ARF are the two most common causes of acquired heart disease in the pediatric population (1–3). ARF is an important complication of β-hemolytic group-A streptococcal infection that may lead to life-long cardiac morbidity primarily due to sequelae of valvular involvement (2). In contrast, KD is a medium vessel vasculitis of unknown etiology that has the predilection to involve coronary arteries and myocardium (3, 81). As we have reviewed, the incidence of ARF is on the decline in majority of the “high-risk” regions. This could be due to general improvement in health and sanitation facilities and effective public health programs. However, robust population-based data on the incidence of ARF are lacking from many high-risk regions with the most notable exception of Northern Australia – the region with the highest incidence of ARF amongst the Indigenous population. Even within Northern Australia, the incidence of ARF is seemingly high in regions where there is stringent data collection (58). Based on this corollary, KD may be under-recognized in the Indigenous population (64, 66). The robust ARF surveillance network may be leveraged to know the incidence of KD specifically amongst the Indigenous population. It would be intriguing to know the epidemiology of KD in populations with the highest incidence of ARF.

The most notable cardiac sequel of KD is the formation of coronary artery aneurysms. These aneurysms may get thrombosed in the acute stage and even lead to myocardial infarction (3). Myocardial involvement (myocarditis), increasingly thought to be universal in KD, may be severe enough to lead to severe cardiogenic shock (81). Long-term cardiac complications of KD include the risk the premature coronary artery disease, early myocardial infarction, and possibly cardiomyopathy (3, 81). It is imperative to mention that the diagnosis of KD may easily be missed especially in developing countries. Lack of awareness, poor healthcare infrastructure, and increased burden of infectious diseases (many of tropical infections may mimic KD) are some of the major contributing factors. The available diagnostic criteria for KD are based on constellation of clinical features and there is lack of a specific diagnostic test. Besides, epidemiological data collection in many developing countries remains suboptimal. These issues can further compound the problem of underdiagnosis of KD in developing countries. Nevertheless, increase in the incidence of KD has still been noted in the developing countries which has been attributed to industrialization and urbanization, besides the increase in the general awareness regarding the disease (82, 83). In the developed world, has already overtaken ARF as the leading cause of acquired heart disease in children. The developing countries also seem to be on a similar trend.

Recognition of KD and ARF in the pediatric population would help to reduce the burden of acquired heart disease in a significant proportion of the economically productive age group. This would have immense implications for the developing countries many of which face significant economic constraints (82). Surveillance networks simultaneously assessing the incidence of KD and ARF would be an effective option to tackle the major chunk of acquired heart disease in children in these regions.

Finally, multisystem inflammatory syndrome in children (MIS-C) post-SARS-CoV-2 usually presents with KD-like features. Myocardial dysfunction is seen in approximately half, whereas, coronary artery involvement is seen in about one-tenth (84). The SARS-CoV-2 pandemic has resulted in a spike in the incidence of KD in many countries around the globe with may further add to the burden of acquired heart disease in children in the near future. On a global stage, especially in the developing countries, the young hearts faced a dual challenge of ARF and KD even before the SARS-CoV-2 pandemic. MIS-C post-SARS-CoV-2 has compounded the challenge of mitigating the problem of acquired heart disease in children. A dedicated collaborative effort is required on a global level to manage the predicament of acquired heart disease in children.

## Author Contributions

AB: writing of initial draft of manuscript, editing and revision of manuscript at all stages of its production, review of literature, drawing of figures, and final approval. SM: writing of initial draft of the manuscript, contributed to editing of manuscript, review of literature, and final approval. PB, AS, and RK: contributed to editing of manuscript, data collection, and final approval. PV: contributed to editing of manuscript, critical revision of the manuscript at all stages of production, review of literature, and final approval. SS: contributed to editing of manuscript, revision of the manuscript, and its final approval. All authors contributed to the article and approved the submitted version.

## Conflict of Interest

The authors declare that the research was conducted in the absence of any commercial or financial relationships that could be construed as a potential conflict of interest.

## Supplementary Material

Supplementary Table 1 American Heart Association (2015) Revised Jones criteria for the diagnosis of acute rheumatic fever (ARF).

Supplementary Table 2 American Heart Association (2017) criteria for diagnosis of Kawasaki disease (KD).

Supplementary Table 3 Japanese criteria (2020) for diagnosis of Kawasaki disease (KD).

Supplementary Table 4 Summary of trends in incidence of acute rheumatic fever in “high-risk” regions.

Supplementary Table 5 Summary of trends in incidence of Kawasaki disease in children below 5 years of age (unless specified otherwise) in regions classified as “high-risk” for acute rheumatic fever.
