# Supplementary material for: What Lies Ahead for Young Hearts in the 21st Century – Is It Double Trouble of Acute Rheumatic Fever and Kawasaki Disease in Developing Countries?
Source: Front Cardiovasc Med. 2021 Jun 24;8:694393. doi: 10.3389/fcvm.2021.694393 (PMC8263915; doi:10.3389/fcvm.2021.694393)
Supplement: Supplementary Table 1 — American Heart Association (2015) Revised Jones criteria for the diagnosis of acute rheumatic fever (ARF). [file Table_1.DOCX]

**Supplementary Table 1:** 2015 American Heart Association Revised Jones criteria for the diagnosis of acute rheumatic fever (ARF).

| Initial episode of ARF is diagnosed in presence of 2 major or 1 major and 2 minor criteria in presence of evidence of group A streptococcal infection in recent past. Recurrent ARF can be diagnosed in presence of 3 minor criteria as well. | | |
| --- | --- | --- |
| 1. Moderate- and high-risk populations | | |
| **A:** | Major criteria | 1. Carditis, including echocardiographic features  2. Arthritis, encompassing polyarthritis, monoarthritis, or (after excluding other causes) polyarthralgia  3. Erythema marginatum  4. Subcutaneous nodules  5. Chorea |
| **B:** | Minor criteria | 1. Monoarthralgia  2. Fever of >100.4°F (>38°C)  3. C-reactive protein of >30 mg/L and/or erythrocyte sedimentation rate of >30 mm in 1^st^ hour  4. Prolonged PR interval on electrocardiography |
| 1. Low-risk populations | | |
| **A:** | Major criteria | 1. Carditis, including echocardiographic features  2. Arthritis, encompassing polyarthritis only  3. Erythema marginatum  4. Subcutaneous nodules  5. Chorea |
| **B:** | Minor criteria | 1. Polyarthralgia  2. Fever of >101.3°F (>38.5°C)  3. C-reactive protein of >30 mg/L and/or erythrocyte sedimentation rate of >60 mm in 1^st^ hour  4. Prolonged PR interval on electrocardiography |

Source: Reference 20
