# Supplementary material for: What Lies Ahead for Young Hearts in the 21st Century – Is It Double Trouble of Acute Rheumatic Fever and Kawasaki Disease in Developing Countries?
Source: Front Cardiovasc Med. 2021 Jun 24;8:694393. doi: 10.3389/fcvm.2021.694393 (PMC8263915; doi:10.3389/fcvm.2021.694393)
Supplement: Supplementary Table 2 — American Heart Association (2017) criteria for diagnosis of Kawasaki disease (KD). [file Table_2.DOCX]

**Supplementary Table 2:** American Heart Association [2017] criteria for diagnosis of Kawasaki disease (KD).

| 1. Complete KD is diagnosed if both criteria **A** and **B** are fulfilled during the clinical course: | | |
| --- | --- | --- |
| **A:** | Fever for >5 days | Although rare, KD experts may be able to diagnose KD after 3–4 days of fever also |
| **B:** | >4 of the following clinical features | 1. Conjunctival injection (non-exudative, bilateral bulbar conjunctiva involved)  2. Orolabial changes (erythema of oropharyngeal mucosa, lips (with cracking); or strawberry tongue)  3. Enlarged cervical nodes of >1.5 cm  4. Pleomorphic rash  5. Erythematous edema of extremities (hands or feet) or periungual skin peeling |
| 1. Incomplete KD is diagnosed when criteria **A**, at least >2 criteria **B**, and criteria **C** are fulfilled. However, incomplete KD can be diagnosed in infants who fulfill criteria **A** and **C** only. | | |
| **C:** | Abnormalities on echocardiography, >1 of the following | 1. Coronary artery aneurysms  2. Left anterior descending or right coronary artery dimensions >2.5 z-score  3. >3 of the following:  i. Left anterior descending or right coronary artery dimensions 2.0–2.5 z-score  ii. Diminished left ventricular function  iii. Pericardial effusion  iv. Mitral regurgitation |
| 1. Treatment for incomplete KD can be initiated when criteria **A**, at least >2 criteria **B**, criteria **D,** and criteria **E** are fulfilled. However, Treatment for incomplete KD can be initiated in infants who fulfill criteria **A**, **D**, and **E** only. These children should be closely monitored for development of manifestations described in criteria **B** and **C**. | | |
| **D:** | >1 of the following | 1. C-reactive protein of >30 mg/L  2. Erythrocyte sedimentation rate of >40 mm in 1^st^ hour |
| **E:** | >3 of the following | 1. Anemia (as per age cut-offs)  2. Total white blood cell (WBC) count >15.0×10^9^/L  3. Platelet count >450×10^9^/L (after day 7 of fever)  4. Serum albumin <30 g/L  5. Elevated alanine aminotransferase  6. WBCs in urine of >10 per high power field |

Source: Reference 3
