# Supplementary material for: What Lies Ahead for Young Hearts in the 21st Century – Is It Double Trouble of Acute Rheumatic Fever and Kawasaki Disease in Developing Countries?
Source: Front Cardiovasc Med. 2021 Jun 24;8:694393. doi: 10.3389/fcvm.2021.694393 (PMC8263915; doi:10.3389/fcvm.2021.694393)
Supplement: Supplementary Table 3 — Japanese criteria (2020) for diagnosis of Kawasaki disease (KD). [file Table_3.DOCX]

**Supplementary Table 3:** Japanese criteria [2020] for diagnosis of Kawasaki disease (KD).

| 1. Complete KD is diagnosed if criteria **A** are fulfilled during the clinical course: | | |
| --- | --- | --- |
| **A:** | >5 of the following clinical features | 1. Fever of any duration  2. Conjunctival injection (bilateral bulbar conjunctiva involved)  3. Orolabial changes (erythema of oropharyngeal mucosa, lips; or strawberry tongue)  4. Enlarged cervical nodes (non-suppurative)  5. Rash including erythema at Bacillus Calmette–Guerin vaccination site  6. Erythema or edema of extremities (hands or feet) or periungual skin peeling |
| 1. Complete KD is diagnosed when abnormalities on echocardiography are noted along with >4 features mentioned in criteria **A**. | | |
| 1. Incomplete KD is diagnosed when abnormalities on echocardiography are noted along with 3 features mentioned in criteria **A** and other causes of fever have been ruled out. | | |
| 1. In absence of abnormalities on echocardiography, incomplete KD can be when 3 or 4 features mentioned in criteria **A** are noted, other causes of fever have been ruled out, and criteria **B** is fulfilled. | | |
| **B:** | Some of the following | 1. Elevated platelet count during convalescent phase  2. Elevation of hepatic transaminases during acute phase  3. Decreased levels of albumin or sodium in the blood  4. Elevated levels of B-type natriuretic peptide (BNP) or N-terminal pro-BNP  5. Echocardiographic findings of pericardial effusion or mitral valve regurgitation  6. Enlargement (hydrops) of the gallbladder  4. In infants, elevated white cells in urine sediment |
| 1. In absence of abnormalities on echocardiography, incomplete KD may be considered when 1 or 2 features mentioned in criteria **A** are noted, other causes of fever have been ruled out, and criteria **B** is fulfilled. | | |

Source: Reference 26
