# Supplementary material for: What Lies Ahead for Young Hearts in the 21st Century – Is It Double Trouble of Acute Rheumatic Fever and Kawasaki Disease in Developing Countries?
Source: Front Cardiovasc Med. 2021 Jun 24;8:694393. doi: 10.3389/fcvm.2021.694393 (PMC8263915; doi:10.3389/fcvm.2021.694393)
Supplement: Supplementary Table 4 — Summary of trends in incidence of acute rheumatic fever in “high-risk” regions. [file Table_4.DOCX]

**Supplementary Table 4:** Summary of trends in incidence of acute rheumatic fever in ‘high-risk’ regions.

| **Serial no., year(s) [reference]** | **Area within the region** | **Age-group** | **Average annual incidence (per 100,000)** |
| --- | --- | --- | --- |
| 1. **India** | | | |
| 1. 1976-78 [27] | Kaniampadi, Vellore (rural) | 4–16 years | 110 |
| 2. 1986 [29] | Anand, Gujarat (rural) | 8–18 years | 38.4 |
| 3. 1987 [29] | Ludhiana, Punjab (combined) | 6–16 years | 70 |
| 4. 1988-91 [28] | Ambala, Haryana (rural) | 5–15 years | 54 |
| 5. 1992-93 [30] | Shimla (combined) | 5–16 years | 32 |
| 5. 1984-94 [29] | Delhi (urban) | 5–10 years | 38.4 |
| 6. 2000 [29] | Kanpur (combined) | 7–15 years | 75 (42 in urban and 120 in rural areas) |
| 7. 2007-08 [29] | Shimla (combined) | 5–16 years | 0 |
| 1. **China** | | | |
| 1. 1993-94 [38] | Sichuan (west area, combined) | 5–18 years | 12.87 |
| 2. 1992-95 [39, 40] | Mainland (5 provinces) | 5–18 years | 20.05 |
| 1. **Western Australia** | | | |
| 1. 1975-79 [51] | West Kimberley | School children (I) | 230–350 |
| 2. 1988-92 [52] | Kimberley region | 5–14 years (I) | 375 |
| 1. **Queensland, Australia** | | | |
| 1. 1999-2004 [55] | North Queensland | 5–14 years (I) | 133 |
| 2. 2004-09 [56] | North Queensland | 5–14 years (I) | 155 |
| 1. **Northern Territory, Australia** | | | |
| 1. 1978-87 [57] | Central Australia | 5–14 years (I) | 815 |
| 2. 1987-96 [58] | Top End Northern Territory | 5–14 years (I) | 224 (508 in areas with complete data) |
| 3. 1979-96 [59] | Central Australia | 5–14 years (I) | 351 |
| 4. 1979-96 [59] | Top End Northern Territory | 5–14 years (I) | 245 |
| 5. 1997-2010 [60] | Northern Territory | 5–14 years (I) | 194 |
| 1. **Australia** | | | |
| 1. 2015-17 [61] | Across 5 jurisdictions | 5–14 years (I) | 107.6 |
| 1. **Africa** | | | |
| 1. 1990 [68] | Tunisia | School children | 30 |
| 2. 1997 [69] | Algeria | 4–19 years | 11.1 |
| 3. 2000 [69] | Algeria | 4–19 years | 6.2 |
| 1. **Latin America** | | | |
| 1. 1982-83 [74] | Martinique, French Caribbean | <20 years | 19.6 |
| 2. 1982-83 [74] | Guadeloupe, French Caribbean | <20 years | 17.4 |
| 3. 1992 [69, 72] | Belo Horizonte, Brazil | 10–20 years | ~360 |
| 4. 1986 [73] | Pinar del Rio, Cuba | 5–25 years | 18.6 |
| 5. 1996 [73] | Pinar del Rio, Cuba | 5–25 years | 2.5 |
| 6. 1979 [75] | Chile | NA | 3 |
| 7. 1998 [75] | Chile | NA | 0 |
| 8. 1970s [77] | Mexico | NA | 1060 |
| 9. 1994-99 [76] | Mexico | 5–20 years | 700 |
