# Supplementary material for: What Lies Ahead for Young Hearts in the 21st Century – Is It Double Trouble of Acute Rheumatic Fever and Kawasaki Disease in Developing Countries?
Source: Front Cardiovasc Med. 2021 Jun 24;8:694393. doi: 10.3389/fcvm.2021.694393 (PMC8263915; doi:10.3389/fcvm.2021.694393)
Supplement: Supplementary Table 5 — Summary of trends in incidence of Kawasaki disease in children below 5 years of age (unless specified otherwise) in regions classified as “high-risk” for acute rheumatic fever. [file Table_5.DOCX]

**Supplementary Table 5:** Summary of trends in incidence of Kawasaki disease in children below 5 years of age (unless specified otherwise) in regions classified as ‘high-risk’ for acute rheumatic fever.

| **Serial no., year(s) [reference]** | **Area** | **Average annual incidence (per 100,000)** |
| --- | --- | --- |
| 1. **India** | | |
| 1. 1994 [33] | Chandigarh | 0.51 (<15 years) |
| 2. 2007 [33] | Chandigarh | 4.54 (<15 years) |
| 3. 2009-14 [34] | Chandigarh | 2.56 (<15 years) |
| 4. 2009-14 [34] | Chandigarh | 5.35 |
| 5. 2015 | Chandigarh | 5.64 |
| 6. 2019 | Chandigarh | 10.6 |
| 1. **China** | | |
| 1. 1998-2002 [42–44] | Shanghai | 27.32 |
| 2. 2003-07 [42–44] | Shanghai | 46.32 |
| 3. 2008-12 [42–44] | Shanghai | 50.5 |
| 4. 2103-17 [42–44] | Shanghai | 94.7 |
| 5. 1995-99 [45] | Beijing | 22.9 |
| 6. 2000-04 [46] | Beijing | 49.4 |
| 1. **China, other regions** | | |
| 1. 1993-97 [49] | Shaanxi | ~2.34 |
| 2. 1997 [50] | Sichuan | 4.26 |
| 3. 2001 [50] | Sichuan | 9.81 |
| 4. 1999-2008 [47] | Jilin province | 5.04 |
| 5. 2001-13 [48] | Inner Mongolia | 3.55 |
| 1. **Australia** | | |
| 1. 1979-90 [62] | Adelaide | 3.9 |
| 2. 1994 [63] | Nationwide study | 3.7 |
| 3. 1979-89 [64] | Western Australia | 2.82 |
| 4. 1989-99 [64] | Western Australia | 7.96 |
| 5. 1999-2009 [64] | Western Australia | 9.34 |
| 6. 2015-16 [65] | Newcastle | ~25 |
| 1. **Africa** | | |
| 1. 2005-08 [71] | Algeria | 3.15 |
| 2. 2001-09 [71] | Morocco | 4.52 |
| 3. 1996-2013 [71] | Tunisia | 0.95 |
| 1. **Latin America** | | |
| 1. 2001-04 [79, 80] | Chile | 5.7 |
| 2. 2005-07 [79, 80] | Chile | 8.4 |
| 3. 2009-2011 [79, 80] | Chile | 10.4 |
